# Insights into the prenatal origin of childhood acute lymphoblastic leukemia

**DOI:** 10.1007/s10555-019-09841-1

**Published:** 2020-01-04

**Authors:** Daniel Hein, Arndt Borkhardt, Ute Fischer

**Affiliations:** Department of Pediatric Oncology, Hematology and Clinical Immunology, University Children’s Hospital, Medical Faculty, Heinrich-Heine-University Düsseldorf, Düsseldorf, Germany

**Keywords:** Prenatal origin, ALL, Preleukemia, Fusion genes

## Abstract

Pediatric acute lymphoblastic leukemia (ALL) is defined by recurrent chromosomal aberrations including hyperdiploidy and chromosomal translocations. Many of these aberrations originate *in utero* and the cells transform in early childhood through acquired secondary mutations. In this review, we will discuss the most common prenatal lesions that can lead to childhood ALL, with a special emphasis on the most common translocation in childhood ALL, t(12;21), which results in the *ETV6-RUNX1* gene fusion. The *ETV6-RUNX1* fusion arises prenatally and at a 500-fold higher frequency than the corresponding ALL. Even though the findings regarding the frequency of *ETV6-RUNX1* were originally challenged, newer studies have confirmed the higher frequency. The prenatal origin has also been proven for other gene fusions, including *KMT2A*, the translocations t(1;19) and t(9;22) leading to *TCF3-PBX1* and *BCR-ABL1*, respectively, as well as high hyperdiploidy. For most of these aberrations, there is evidence for more frequent occurrence than the corresponding leukemia incidences. We will briefly discuss what is known about the cells of origin, the mechanisms of leukemic transformation through lack of immunosurveillance, and why only a part of the carriers develops ALL.

## Introduction

**Fig. 1:**
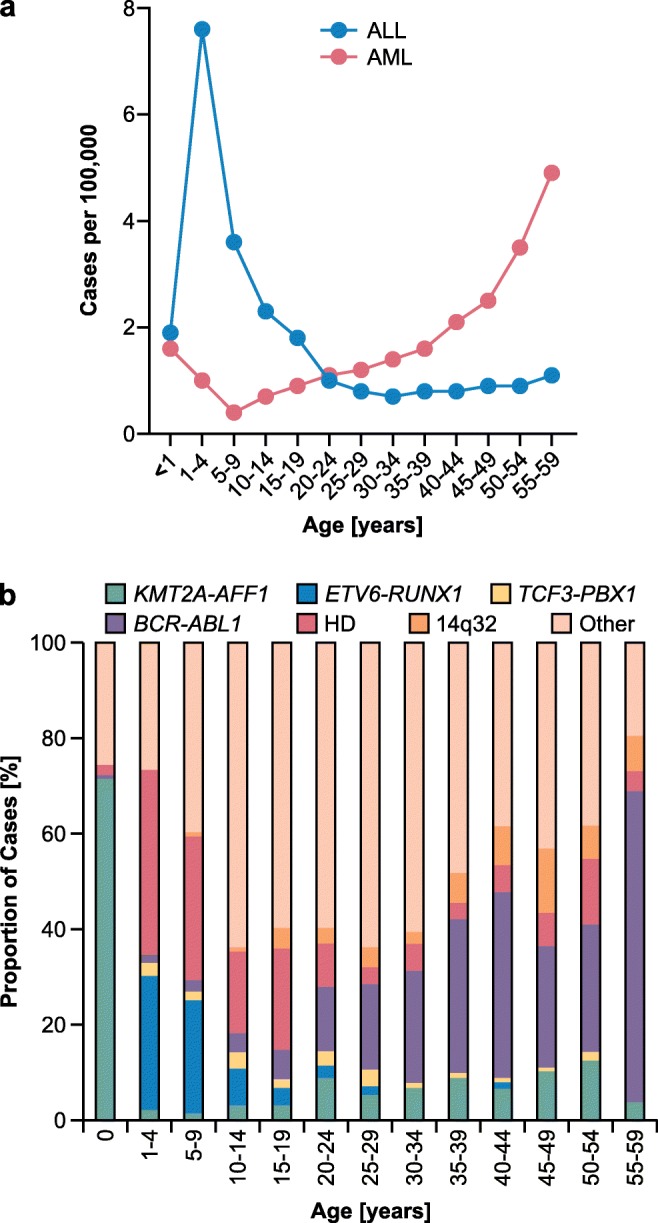
Age distribution and major subtypes of ALL. **a** Age distribution of ALL and AML in the USA from 1975 to 2016. Cases per 100,000 are shown. ALL has a clear peak at ages 1–4 and 5–9, whereas AML rates rise with age. **b** Major subtypes of ALL divided by age groups. The *KMT2A-AFF1* fusion is very prevalent in infants, *ETV6-RUNX1* and high hyperdiploidy (HD) dominate childhood ALL, and *BCR-ABL1* is the most prevalent aberration in adults. Data for (**a**) taken from [1], data from (**b**) taken from [3]

Acute lymphoblastic leukemia (ALL) is the most common leukemia subtype in children [1]. The vast majority of cases belong to the B cell precursor subtype, whereas roughly 15% carry T cell progenitor markers [2]. ALL is common in young children and incidence peaks at ages 2–5 years. This peak is absent from acute myelogenous leukemia (AML), which is more common in adults (Fig. 1a) [1]. Infant ALL with *KMT2A* (previously known as *MLL*) rearrangements being a possible exception, childhood ALL is caused by a combination of genetic susceptibility factors and subsequently acquired somatic mutations. These mutations often occur in genes that are critical for lymphoid development [4]. The genetic susceptibility factors are often recurrent nonrandom mutations, like translocations or hyperdiploidy. These factors correspond to the ALL group in which they appear and can be used to classify the ALL. Depending on the age of the patients, different mutations are predominant. For infants, *KMT2A* rearrangements are the most common aberration; in young children, high hyperdiploidy and the translocation t(12;21), causing *ETV6-RUNX1* (*TEL-AML1*), are most common (Fig. 1b) [2]. Another common translocation is t(1;19), leading to *TCF3-PBX1* (*E2A-PBX1*), which occurs in approximately 5% of childhood cases as well as in adult ALL [5]. The *BCR-ABL1* fusion, as a result of a t(9;22), is relatively rare in childhood ALL but is the most common aberration in adult ALL (Fig. 1b) [5].

**Fig. 2:**
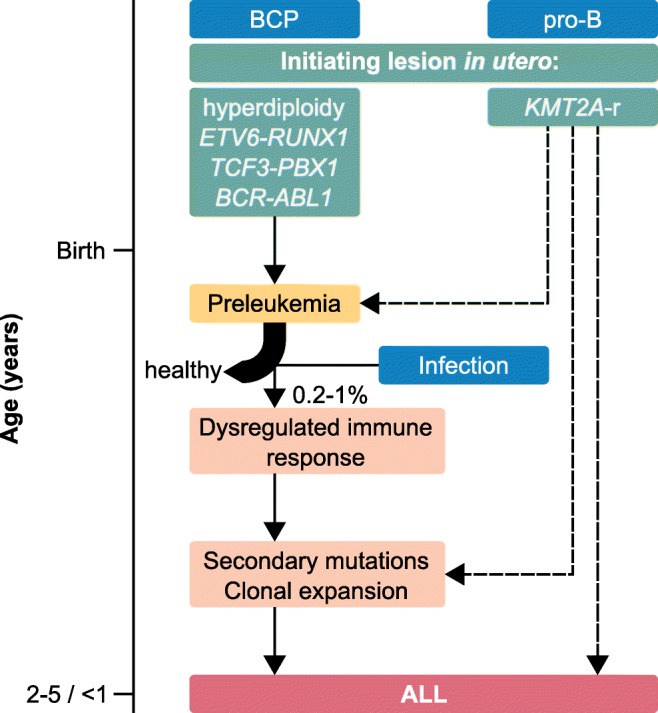
Timeline of ALL development. The initiating lesions (green) occur *in utero* and lead to a state of preleukemia after birth. Exposure to infection leads to a dysregulated immune response in a small fraction (about 0.2–1%) of preleukemic children. Most preleukemic children remain healthy [6, 7]. The children develop ALL by acquiring secondary mutations, eventually leading to clonal expansion. In the case of *KMT2A* rearrangements (*KMT2A*-r, right), it is not completely clear whether the *KMT2A*-r are sufficient for ALL development (right dashed arrow). It is also possible that the *KMT2A*-r directly trigger secondary mutations (central dashed arrow). One case of a healthy *KMT2A*-r carrier has been reported [13], leading to the possibility of a preleukemic state for those cases (left dashed arrow). The given median ages of ALL onset are 2–5 years of age for B cell precursor subtypes (BCP, left) and < 1 year of age for pro-B cell subtype (pro-B, right)

As secondary alterations are needed for most subtypes, these first hits produce a preleukemic state. The secondary mutations only occur in a fraction of carriers [6–9]. There is convincing evidence that a significant percentage of these preleukemic lesions can arise prenatally and transform after postnatal secondary events occur [6–12] (Fig. 2). This evidence comes primarily from twin studies in which both twins had identical breakpoints, immunoglobulin heavy chain (IgH), or T cell receptor (TCR) rearrangements [10, 11, 14–16]. Additionally, by tracing the leukemia back to neonatal blood spots (Guthrie cards) of the twins, studies have been able to generate further proof of prenatal origin [17–19]. Here, the identical breakpoints were also present on the Guthrie cards. Since the blood is taken immediately after birth, a postnatal origin can be ruled out. The first hit occurs in the cell of origin, which differs from subtype to subtype. *KMT2A* rearrangements probably occur at the earliest state in CD34^+^ and CD19^−^ cells [20], while other translocations seem to arise later in B cell development, although the exact cell is usually unknown. Infection [21] and delayed infection [22] have been discussed as possible causes for leukemic transformation for over a century. Furthermore, the mixing of populations has been postulated as a causal factor for leukemic transformation [23]. Recent studies have shown that exposure to infection can trigger the progression from preleukemia to ALL [24, 25]. A dysregulated immune response by activation of preleukemic B cells through memory T helper cells [26] and inactivity of NK cells have also been discussed [27–29].

To date, the prenatal origin of ALL with *KMT2A* rearrangements [10], *ETV6-RUNX1* [11], *TCF3-PBX1* [9], *BCR-ABL1* [8], and (high) hyperdiploidy [12] has been shown (Table 1).

**Table 1:** Studies that identified prenatal ALL or preleukemia

Subtype	Year	Study	Specimen	Positives (prevalence)	Study type	Method of detection	Population	Preleukemic cells
*KMT2A*-r	1993	Ford et al. [10]	Bone marrow, peripheral blood, testicles	6/6 (100%)	LTS	Southern Blotting	N/S	
	1994	Gill Super et al. [15]	Peripheral blood	2/2 (100%)	LTS	Southern Blotting	N/S	
	1997	Gale et al. [17]	Guthrie cards	3/3 (100%)	BT	PCR	N/S	
	1998	Uckun et al. [13]	Fetal bone marrow, fetal liver	9/29 (31.03%)	PNS	nRT-PCR	N/S	
	2000	Yagi et al. [12]	Guthrie cards	2/2 (100%)	BT	nPCR	N/S	
	2000	Fasching et al. [30]	Guthrie cards	2/2 (100%)	BT	PCR	Austrian	
	2002	Taub et al. [31]	Guthrie cards	1/1 (100%)	BT	PCR	US American	
*ETV6-RUNX1*	1998	Ford et al. [11]	Guthrie cards	2/2 (100%)	LTS/BT	PCR	Dutch	
	1999	Wiemels et al. [14, 18]	Guthrie cards	8/11 (72.73%)	BT	LDI-PCR	British, Italian	
	2001	Maia et al. [19]	Guthrie cards	3/3 (100%)	BT	PCR	N/S	
	2001	Eguchi-Ishimae et al. [32]	Cord blood	1/67 (1.49%)	NBS	nRT-PCR	Japanese	
	2002	Mori et al. [6]	Frozen cord blood	6/567 (1.06%)	NBS	nRT-PCR qRT-PCR FISH	British	10^−3^ to 10^−4^
	2002	Taub et al. [31]	Guthrie cards	1/1 (100%)	BT	PCR	US American	
	2006	Burjanivova et al. [33]	Guthrie cards	1/3 (33.33%)	BT	PCR	Czech	
	2008	Gruhn et al. [34]	Guthrie cards	3/6 (50.00%)	BT	snPCR	German	10^−4^ to 10^−5^
	2011	Zuna et al. [35]	Cord blood	5/253 (1.98%)	NBS	RT-PCR, qRT-PCR	Czech	
	2012	Olsen et al. [36]	Fresh cord blood	3/1258 (0.24%)	NBS	qRT-PCR	Danish	< 10^−4^
	2014	Škorvaga et al. [37]	Frozen cord blood	8/200 (4.00%)	NBS	qRT-PCR	Slovak	≤ 10^−5^
	2015	Ornelles et al. [38]	Fresh cord blood	5/210 (2.38%)	NBS	nRT-PCR	US American	
	2017	Kosik et al. [39]	Cord blood	4/300 (1.33%)	NBS	qRT-PCR	Slovak	≤ 10^−5^
	2018	Schäfer et al. [7]	Frozen cord blood	50/1000 (5.00%)	NBS	GIPFEL	Danish	10^−2^ to 10^−5^
*TCF3-PBX1*	2002	Wiemels et al. [40]	Guthrie cards	2/15 (13.33%)	BT	PCR	US American	
	2002	Taub et al. [31]	Guthrie cards	1/1 (100%)	BT	PCR	US American	
	2019	Hein et al. [9]	Frozen cord blood	2/340 (0.59%)	NBS	GIPFEL	Danish	10^−3^ to 10^−4^
*BCR-ABL1*	2011	Cazzaniga et al. [8]	Bone marrow, peripheral blood, Guthrie cards	4/4 (100%)	LTS/BT	RT-PCR, qRT-PCR, FISH	Italian, British	
Hyperdiploid	2000	Yagi et al. [12]	Guthrie cards	1/1 (100%)	BT	nPCR	N/S	
	2002	Panzer-Grümeyer et al. [41]	Guthrie cards	1/1 (100%)	BT	nPCR	Austrian	
	2002	Taub et al. [31]	Guthrie cards	5/5 (100%)	BT	PCR	US American	
	2003	Maia et al. [16]	Bone marrow, peripheral blood	2/2 (100%)	BT	nPCR	British	
	2008	Gruhn et al. [34] (including Taub et al. [31])	Guthrie cards	10/11 (90.91%)	BT	snPCR	German, US American	10^−4^
Other	2000	Yagi et al. [12]	Guthrie cards	1/4 (25.00%)	BT	nPCR	N/S	
	2002	Taub et al. [31]	Guthrie cards	4/9 (44.44%)	BT	PCR	US American	
	2006	Burjanivova et al. [33]	Guthrie cards	2/9 (22.22%)	BT	PCR	Czech	
	2008	Gruhn et al. [34]	Guthrie cards	11/20 (55.00%)	BT	snPCR	German	10^−3^ to 10^−5^

The number of preleukemic cells is only given for studies that investigated preleukemia and determined its frequency. Frequencies for studies that used GIPFEL are from sorted CD19^+^ cells

*KMT2A-r*, cases with *KMT2A* rearrangements; *other*, not specified ALL cases; *LTS*, leukemic twin study; *BT*, backtracking study; *PNS*, prenatal screening study; *NBS*, newborn screening study; *N/S*, not specified

## Prenatal subtypes

### *KMT2A* rearrangements

The *KMT2A* gene has a myriad of fusion partners. For ALL, the most important one is surely *AFF1*, with *MLLT3* (*AF9*) and *MLLT1* (*ENL*) also being more frequent than others [5]. *KMT2A* translocations frequently occur in infants, and some newborns already show signs of full-blown leukemia [15]. Therefore, these translocations are a natural candidate for prenatal development. Among several studies that investigated the prenatal status of ALL in general and *KMT2A*-rearranged ALL in particular, were those that first traced ALL back to an *in utero* event [10, 15] and the first to trace it back to Guthrie cards [17]. Uckun et al. [13] showed actual *in utero* presence of the fusion gene in fetal tissue from abortions. This study also found one case of a healthy infant expressing the *KMT2A-AFF1* fusion transcript. This suggests that *KMT2A* fusions are also present in healthy individuals and will not necessarily lead to overt leukemia. However, it is unknown whether this infant developed leukemia later on. Additionally, other studies [42, 43] failed to reproduce these findings, leaving the question of whether *KMT2A* translocations also occur more frequently than the corresponding leukemia at least in part unanswered. It is of note, though, that one should expect to find *KMT2A* fusions in fetal tissue, even if leukemia development was inevitable. For that to happen, the cohort size would simply have to be much larger than the 29 samples studied by Uckun et al. [13].

There are two possibilities that explain how the *KMT2A* fusions contribute to leukemia development and the short latency periods after birth: (1) The fusion itself is sufficient for leukemia onset. This would mean that leukemia development is inevitable and no healthy individuals carrying the fusions exist. (2) A secondary mutation is required, but is triggered by the fusion protein. This would also be in line with the short latency. If the fusions trigger the additional mutation, leukemia development might be inevitable, but it would allow for the theoretical possibility of healthy carriers (Fig. 2). As only one such case has been described [13], it is not possible to infer from the presence or absence of healthy carriers which model is actually at work.

### *ETV6-RUNX1*

There is ample evidence that the translocation t(12;21) leading to the fusion of the transcription factors *ETV6* and *RUNX1* predominantly, maybe even always, arises *in utero* [11, 18]. This was first shown in a twin study in 1998 [11]. Here, both twins had exactly the same breakpoint, something that had never before been described for *ETV6-RUNX1*^+^ leukemia [11]. This supports a model in which the preleukemic clone arises in one twin and spreads to the other *via* the shared placenta. The secondary mutations in the twins differed, hinting at postnatal origin. Additionally, this and several other studies were able to trace back this leukemia type to Guthrie cards [11, 18, 19, 31, 33, 34].

The *ETV6-RUNX1* fusion alone is not sufficient for leukemia development. For that, secondary postnatal mutations are necessary. Therefore, not without controversy regarding the frequency of the translocation, several studies have investigated the *ETV6-RUNX1* fusion in healthy individuals, especially newborns. Initially identified in umbilical cord blood of one healthy newborn and the peripheral blood of 13 healthy children and adults [32], *ETV6-RUNX1* was shown to be present in ~ 1% of newborns [6]. Several Danish studies later challenged these findings [36, 44–47], but newer reports confirmed the original results [7, 37–39].

There are several possible explanations for the contradicting results of these studies. The material used is one of the factors that can influence the outcome. All studies used umbilical cord blood (UCB) for the investigation of newborns. However, some studies used fresh UCB, handled within 24 h of blood draw, while others used frozen UCB or did not specify whether the material was fresh. Interestingly, all studies that identified no or very few *ETV6-RUNX1*^+^ cells in the UCB used fresh UCB [45, 46, 48] or in one case fresh embryonic liver [44]. Using freshly harvested cells has the advantage of accurately representing the neonatal hematopoietic environment. It does, however, require a great deal of time and money. It is unlikely that the different results are influenced by the use of fresh or stored UCB, because (1) one study by Ornelles et al. [38] used fresh UCB and identified 2.38% *ETV6-RUNX1*^+^ samples, and (2) storage has a negative effect on RNA, especially when RNA is released from dead cells [49], and therefore the studies using frozen UCB should have found fewer *ETV6-RUNX1*^+^ cells. Then again, it was shown that apoptotic signals can induce double-strand breaks in both *ETV6* and *RUNX1* and that this can lead to the *ETV6-RUNX1* fusion [32]. Storage therefore could induce the translocation but probably at very low levels and in much fewer samples than reported by the studies using frozen UCB [6, 7, 37]. Also, if the freezing induced the *ETV6-RUNX1* fusion, Ornelles et al. [38] should not have found any positive samples.

A more likely cause of the different results is the use of different detection methods. Most studies used nested reverse transcriptase PCR (nRT-PCR) or quantitative RT-PCR (qRT-PCR). The advantage of qRT-PCR is that it allows for quantification of the fusion transcript. The nRT-PCR may be more sensitive, as it uses a nested PCR setup, but it is not quantitative. Both methods are, like all RNA methods, vulnerable to contamination, the nRT-PCR approach even more so as it is an open-tube technique. However, contaminations in qRT-PCR can also lead to overestimation of prevalence. However, almost all studies regardless of results used qRT-PCR, and some used multiple techniques for validation. Mori et al. [6] used nRT-PCR and then qRT-PCR and FISH to validate their finding that ~ 1% carried the fusion. Lausten-Thomsen et al. [46] initially found 14 of 1417 (~ 1%) samples to be *ETV6-RUNX1*^+^ by qRT-PCR. After dot-blot validation, nine positives remained. It was only the second validation with RNA of flow-cytometric-sorted frozen UCB cells that led the authors to conclude that the results were falsely positive. Hence, the specific method used may positively or negatively impact the detection of the *ETV6-RUNX1* fusion, in combination with the quality and quantity of the input material. Low-quality or -quantity input material might lead to false-negative results. Ultimately, all studies but one used RNA as basis for their analysis. DNA is more stable than RNA by a factor of 10,000 when stored frozen [49] and is thus the better choice for stored material. Furthermore, RNA produces the same fusion point for every breakpoint. This is advantageous for screening purposes but makes identification of contaminants impossible. Identical breakpoints on the DNA level have only been reported for identical twins [11]. Hence, a possible contamination is easy to detect. To date, we have conducted the only study identifying *ETV6-RUNX1*^+^ cells *via* DNA quantification [7]. We used the novel GIPFEL technique [50], allowing for the indirect identification of chromosomal translocations at the DNA level. In this study, we identified 5% of healthy newborns to be *ETV6-RUNX1*^+^. Additionally, we sequenced the chromosomal breakpoints of five positive samples.

One could argue that differences between populations might lead to different *ETV6-RUNX1* frequencies in the healthy population. Population differences have been identified for some tumor entities, including *ETV6-RUNX1*^*+*^ and *TCF3-PBX1*^+^ leukemias, the latter of which is more frequent in Latin America [51, 52]. *ETV6-RUNX1* is much less common in East Asians [53], Hispanics [54], and especially in Maori, where only 5.4% of pediatric ALL cases harbor this translocation [55]. Interestingly, the survival rates of *ETV6-RUNX1*^+^ Maori did not differ from those of other ethnicities, probably due to equal access to ALL treatment for all in New Zealand [55]. Except for the Japanese study by Eguchi-Ishimae et al. [32] and the US-American study by Ornelles et al. [38], all studies used primarily Caucasian European populations. Therefore, an influence of the population on the frequency of *ETV6-RUNX1* is highly unlikely.

Notably, all studies that could not identify *ETV6-RUNX1*^+^ newborns were conducted with a Danish population. However, Olsen et al. [48] found 10 out of 2005 healthy adults to express *ETV6-RUNX1* at low levels. That is statistically more than would be expected if the incidence were equal to the leukemia rate (*t* test, *P* = 0.0019). This implies that adults carry the fusion at a higher prevalence than the leukemia rate. Therefore, it is safe to assume that the same is also true for children, even though it is not a proof of prenatal origin. Furthermore, we also screened UCB samples from Denmark and we were able to identify *ETV6-RUNX1* carriers [7]. Hence, it is highly unlikely that the differences between the studies are a result of using samples from the Danish population, especially as the leukemia incidence in Denmark does not differ from the incidences of other European countries [56].

The real discussion might not be whether the *ETV6-RUNX1* fusion is present in healthy newborns but at what frequency. Originally, Mori et al. [6] reported frequencies of 10^−4^ to 10^−3^, but those frequencies were not confirmed by later studies [36, 37, 39, 46, 48]. The frequency in investigated adults was markedly lower, but that is in line with the reduced risk for *ETV6-RUNX1*^+^ leukemia in adults [48]. However, all studies confirming the presence of *ETV6-RUNX1* in healthy newborns that looked at the frequency found it to be much lower [37, 39]. Lausten-Thomsen et al. [46] initially found ~ 1% of *ETV6-RUNX1*-positive samples with a frequency of ≤ 10^−5^, therefore this study is very important in challenging the proposed frequency of the preleukemic cells. We also tried to address this in our study [7], but the frequency we found can only be compared with the others under reserve. We used CD19^+^-sorted cells and had a bias, because not all PCR products are amplified in the same way. Therefore, these numbers should be considered an estimate. Furthermore, we used DNA instead of RNA, so this must be taken into account when comparing the studies. In our study, we also confirmed the presence of *ETV6-RUNX1* by qRT-PCR in two cases [7]. The frequency was ~ 10^−4^, which would be more in line with the studies that found low frequencies.

### *TCF3-PBX1*

The *TCF3-PBX1* fusion is the product of a balanced or unbalanced t(1;19) translocation and is among the most frequent aberrations in childhood ALL. It is especially common in Latin America [51, 52] and among black children [57], where as many as 11.8% of childhood ALL cases carry this fusion.

Unlike the aforementioned translocations, *TCF3-PBX1* has long been considered to only arise postnatally. Still, the fusion could be traced back to Guthrie cards by Wiemels et al. in two cases [40]. In both cases, only one segment of the blood spot was positive for the fusion and the fusion points showed signs of site specificity and of terminal deoxynucleotidyl transferase activity, and so *TCF3-PBX1* was declared postnatal. The site specificity hints at aberrant V(D)J recombination. During fetal hematopoiesis, none or few nontemplate nucleotides are inserted, whereas this insertion is common in children and adults [58–60]. However, in another backtracking study, one *TCF3-PBX1* patient could be traced back to the respective Guthrie card by screening for IgH rearrangements [31].

Following our success with GIPFEL and *ETV6-RUNX1* [7], we also looked for *TCF3-PBX1* in healthy newborns. In 2 of 340 (0.6%) cases, we were able to identify the fusion and also the exact fusion point [9]. The presence of the *TCF3-PBX1* fusion in the UCB of newborns is definite proof of prenatal origin. It is, however, not clear if *TCF3-PBX1*^*+*^ newborns will remain healthy throughout their lifetime. In the *ETV6-RUNX1* study [7], 50/1000 (5%) were translocation positive, which gave the study enough statistical power to conclude that most of them will never develop leukemia. For *TCF3-PBX1*, it is unlikely that both newborns will develop ALL but not impossible. Identifying the *TCF3-PBX1* fusion in healthy newborns could prove that *TCF3-PBX1* can arise prenatally but not that the frequency definitely exceeds the ALL incidence. The data from these studies paint a picture in which *TCF3-PBX1* can arise prenatally but possibly also throughout an individual’s lifetime. Studies investigating the frequency of *TCF3-PBX1*^+^ ALL in children and adults found a slight decrease from 5% of ALL cases in children to 3% in adults [5]. Thus, either *TCF3-PBX1* (1) always arises prenatally and can have a very long latency phase, or (2) it can also arise postnatally, explaining the mild decrease from childhood to adulthood.

### *BCR-ABL1*

The *BCR-ABL1* fusion is the product of a t(9;22) translocation, widely known as the Philadelphia chromosome, which was the first ever to be described [61]. The fusion of these genes can create three different proteins: p190, p210, and p230. Each of these differ in their *BCR* breakpoints, with m-BCR (minor) leading to p190, M-BCR (major) to p210, and μ-BCR (micro) to p230 [62, 63]. Classically, *BCR-ABL1* is present in adult chronic myelogenous leukemia (CML), where 90–95% carry the Philadelphia chromosome. Of these patients, over 99% express the p210 isoform. However, the fusion is also present in ALL. In the adult form, 25% have a t(9;22) [5], with the majority also expressing the p210. In this entity, the p190 isoform is also prominently present. In pediatric ALL, *BCR-ABL1* plays a minor role, with only 3% of cases being positive for this fusion [2]. It is of interest, though, that the p190 isoform is the predominant form in pediatric ALL, with 90% expressing this protein.

The p190 isoform was shown to arise prenatally in at least two pairs of monozygotic twins [8]. In both cases, both twins had the identical breakpoint, indicative of prenatal origin. Also, in one twin pair, the fusion could be traced back to the respective Guthrie cards. Interestingly, in one pair of twins, only one twin developed leukemia [8]. It is, of course, possible that the second twin developed ALL later in life, but it shows that secondary hits are necessary and that these hits are acquired postnatally. This also hints at the possibility that *BCR-ABL1* may arise prenatally in children who will never develop ALL. Additionally, the specificity of the isoforms, regarding the resulting leukemia subtype, indicates that the p210 isoform probably arises postnatally, especially when one considers that it is typical for CML, which usually arises later in life.

### IgH or TCR rearrangements

Leukemia can be traced back to birth using not only gene fusions but also rearrangements of the IgH or the TCR. Several studies have used this approach to investigate ALL cohorts [12, 16, 31, 33, 34, 41] (Table 1). Using IgH or TCR rearrangements has the advantage of enabling the study of leukemia entities without a defining gene fusion. In this way, hyperdiploid ALLs have been shown to arise prenatally [12, 16, 31, 34, 41]. Moreover, it is not surprising that the prenatal origin is not restricted to only ALL with fusion genes. In fact, prenatal origin has been shown for roughly two-thirds of childhood ALL subforms [2, 8–12]. One might argue that prenatal origin is not restricted to those subforms but can also occur in other subgroups, as it is also not restricted to ALL. Prenatal origin of childhood leukemia could also be shown for AML [6] but seems to be more common in ALL [33].

## Mechanisms of transformation

Some details remain unknown, even after the origin of a particular ALL is proven to be prenatal, such as the cell of origin or the mechanisms by which this prenatal preleukemia turns into postnatal ALL.

The cell of origin depends on the underlying lesion. Even though shown in leukemic cases that were not traced back to birth, *BCR-ABL1* fusions can have different cells of origin, depending on the isoform. The p210 form originates in hematopoietic stem cells, whereas the p190 form, typical for childhood ALL, originates in a B cell progenitor [64]. For *ETV6-RUNX1*, there is also evidence that the cell of origin is CD34^+^ and CD19^+^, hinting at a more differentiated lymphoid-committed precursor [64–66]. However, there is also evidence for the opposite. Experiments in zebrafish showed that *ETV6-RUNX1* expression restricted to lymphoid cells was incapable of inducing leukemia, whereas expression in all linages led to ALL at a low level, comparable with the human situation [67]. Mice in which the *ETV6-RUNX1* fusion was put under the control of the IgH promoter did not develop leukemia [68], whereas mice developed ALL when the entire bone marrow was transplanted [69], hinting at a cell of origin with an earlier developmental stage. *TCF3-PBX1* fusions also seem to originate at later stages in a more differentiated, lymphoid-committed progenitor [70]. Translocations including the *KMT2A* gene, however, seem to originate earlier, in a CD34^+^, CD19^−^ cell [20]. Importantly, it is generally difficult to determine the exact cell of origin for the ALL subtypes. The leukemic lesions can alter the cell of origin so that its properties change. It is therefore also possible that the preleukemic cell moves—at least in part—backwards in its evolution, so that it eventually shows surface markers and properties of a cell that is hierarchically upstream from the cell of origin. Similarly, it has been postulated that the initiating lesion, e.g., a fusion gene, does not arise in a committed cell but a hematopoietic stem/progenitor cell, which is then reprogrammed by this oncogene. This reprogramming then leads to epigenetic changes that are inherited by the daughter cells [71]. In this model, the oncogene might not even be needed in the actual tumor but would only act in the cell of origin in a hit and run manner [72].

Progression from preleukemia to overt leukemia might differ in the various prenatal subtypes. As mentioned in the *KMT2A* section, these translocations have a very short latency period and may inevitably lead to ALL development. However, one case of a healthy carrier has been reported [13]. For other translocations, healthy carriers have been described [6–9, 35], suggesting that the translocation itself is insufficient for ALL development. *KMT2A* fusions might be sufficient by themselves or actively trigger a secondary mutation, whereas *ETV6-RUNX1*, *TCF3-PBX1*, and *BCR-ABL1* require independent secondary events for transformation. These secondary events often target genes that are important for immune cells in general or B cells in particular. The complete or partial deletion of *IKFZ1* is a common secondary aberration in *BCR-ABL1*^+^ cases and confers a dismal prognosis [8]. Deletions of the second *ETV6* allele have been reported in up to 70% of *ETV6-RUNX1*^+^ cases [73]. *PAX5* and *CDKN2A* are deleted in 28% and 26% of cases, respectively [73]. These deletions in *ETV6-RUNX1*^+^ patients are predominantly caused by aberrant RAG1/2 activity [74]. The fact that only a part of the translocation carriers suffers secondary mutations and the resulting ALL hints at the involvement of environmental factors in acquiring said mutations. A natural candidate for a possible cause of acute leukemia is infection. This was proposed as early as 1917 [21]. Additional alterations to this hypothesis have been made, including delayed infection [22] and population mixing [23]. Recent studies have provided *in vivo* evidence by linking ALL in *Pax5*-heterozygous mice [24] and *ETV6-RUNX1*^+^ mice [25] to exposure to infection. The exact mechanisms of leukemic transformation through infection are not yet fully understood, but dysregulation of the immune system may play a major role. It has been proposed that memory T helper cells can support preleukemic B cell precursors in the bone marrow, and thus support ALL development [26].

Natural killer (NK) cells are also interesting candidates, as they not only play a role in distinguishing between self and foreign but also play an important role in cancer control [75]. One task of NK cells is it to kill aberrant cells. The interaction between killer immunoglobulin-like receptors (KIRs) on NK cells and human lymphocyte antigen (HLA) class I ligands regulates the activity of NK cells. Both, KIRs and HLA, are polymorphic. HLA-C alleles, which are considered to be the dominant KIR ligands, can be grouped as C1 and C2. The KIR haplotypes on chromosome 19q13.4 are A and B. The A-haplotype harbors more inhibitory KIRs, whereas the B-haplotype has more activating KIRs [28]. For both, there is a telomeric and a centromeric cluster. The presence of more activating KIRs seems to increase the risk of ALL development [29]. Furthermore, it has been shown that in patients with positive minimal residual disease, the activating KIR2DS1 receptor was more present, whereas the inhibitory receptors KIR2DL1 and KIR3DL1 were less frequent [28]. In general, a higher frequency of activating KIRs, which are mostly encoded by the B-haplotype, seems to confer a greater risk of developing ALL [29], with the risk coming mainly from the telomeric B-motifs cluster [28]. The elevated risk is due to the fact that a greater number of inhibitory receptors on NK cells leads to a stronger responsiveness and the absence of inhibitory receptors leaves the cells hyporesponsive [76, 77]. Furthermore, an increase in activating signals leads to a decrease in signal intensity over time [78, 79]. The use of the HLA-C allele also has an influence on susceptibility to ALL. The C2 epitope leads to an elevated risk of ALL and a late relapse, whereas C1 homozygosity has a protective effect [27]. The binding of HLA-C2 ligands to KIR2DS1 induces tolerance and renders the NK cells hyporesponsive [80, 81].

**Fig. 3:**
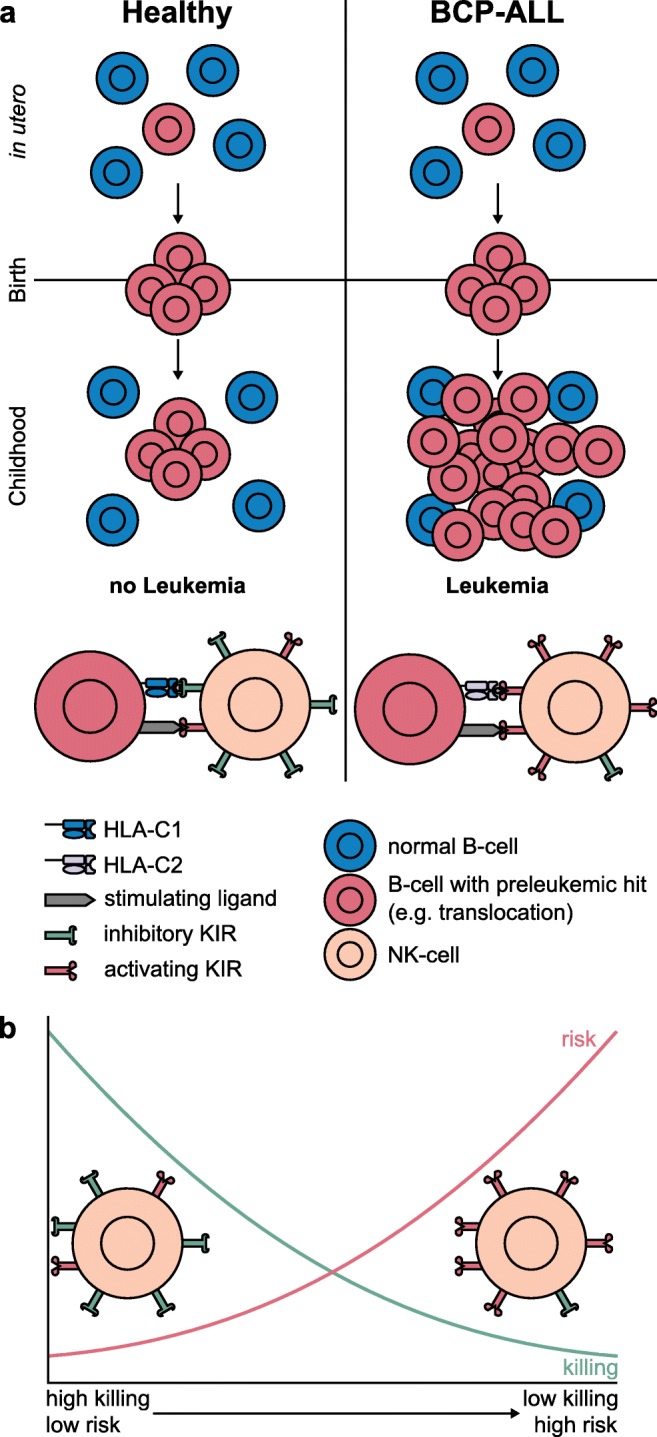
Model of the impact of NK cells on ALL development. **a** A prenatal preleukemic hit emerges *in utero* (red) and an expanded clone is present at birth. According to this model, interaction with NK cells plays a key role in leukemia development. HLA-C2 receptors (lavender) pose an elevated risk for ALL and can also interact with activating KIRs, e.g., KIR2DS1 [82]. An elevated number of activating KIRs (red), especially from the telomeric B cluster also pose a risk (right), whereas HLA-C1 (blue) and more inhibitory KIRs, i.e., the A-haplotype (green) seems to protect against ALL (left). **b** Scheme of NK cell killing efficiency and risk of developing ALL. NK cells with more inhibitory KIRs (green) have a higher killing efficiency and confer a lower risk of ALL; NK cells with more activating KIRs (red) have a lower killing efficiency and confer a higher risk of ALL

Taken together with the data from the prenatal lesions and the fact that these often only induce preleukemia, it is reasonable to assume that the use of HLA-C epitope and KIRs influences not only the likelihood of leukemic transformation (Fig. 3) but also the process itself. If the NK cells fail to clear the preleukemic or leukemic cells, the onset of ALL is more likely or inevitable, respectively. Therefore, among those children in whom the preleukemia progressed to overt leukemia, there should be a higher percentage of HLA-C2 and activating KIRs, whereas among those who are healthy, HLA-C1 and inhibitory KIRs should be predominant.

## Conclusion

Most carriers of prenatal lesions will remain healthy throughout their lifetime. This emphasizes the need for a secondary mutation and possibly a dysregulated immune system. On the other hand, it opens up new possibilities for prevention and treatment. If environmental and genetic factors play a role in leukemic transformation, this offers opportunities for prevention and interference. For instance, if a prenatal lesion is diagnosed, one could also check for HLA-C epitopes and KIR haplotypes to assess the risk of ALL development. A better understanding of the mechanisms of progression from preleukemia to leukemia and why it only happens in a fraction of cases will not only help to treat the respective ALLs but ultimately help to prevent them from occurring in the first place.
